# Multiple rib and vertebral fractures associated with *Bordetella pertussis* infection: a case report

**DOI:** 10.1186/s12879-023-08189-w

**Published:** 2023-04-06

**Authors:** Jingqiao Wang, Junxiang Gao, Hongwei Fan, Haonan Guo, Zundong Yin, Mei Dong, Xiaoming Huang

**Affiliations:** 1 Peking Union Medical College, Chinese Academy of Medical Science, No. 9 Dongdansantiao, Dongcheng District, Beijing, 100730 China; 2 Department of Endocrinology, Union Medical College Hospital, Chinese Academy of Medical Science, No. 1, Shuaifuyuan, Wangfujing Street, Dongcheng District, PekingBeijing, 100730 China; 3 Department of Infectious Diseases, Peking Union Medical College Hospital, Chinese Academy of Medical Science, No. 1, Shuaifuyuan, Wangfujing Street, Dongcheng District, Beijing, 100730 China; 4 Ordos Prefectural Center for Disease Control and Prevention, Ordos, 017000 Inner Mongolia China; 5 Chinese Center of Disease Control and Prevention, National Immunization Program, 155 Changbai Road, Changping District, Beijing, 102206 China; 6 Institute for Immunization and Prevention, Beijing Center for Disease Control and Prevention, No.16 Hepingli Middle Street, Dongcheng District, Beijing, 100013 China; 7 Department of General Internal Medicine, Peking Union Medical College Hospital, Chinese Academy of Medical Science, No. 1, Shuaifuyuan, Wangfujing Street, Dongcheng District, Beijing, 100730 China

**Keywords:** *Bordetella pertussis*, Chronic cough, Rib fractures, Vertebral fractures, Case report

## Abstract

**Background:**

Pertussis is a highly contagious respiratory disease caused by the bacterium *Bordetella pertussis*, characterized by paroxysms of severe coughing, and predominantly affects children. We report the first case of multiple fractures in the ribs, lumbar spine, and sacrum associated with severe coughing caused by *Bordetella pertussis* infection in an adult.

**Case presentation:**

A 49-year-old female presented with acute-onset chest wall pain for 3 weeks. Imaging results revealed multiple fractures in the ribs and vertebrae, as well as bilateral pleural effusion, pericardial effusion, right pneumothorax, and enlargement of the left parapharyngeal and subclavian lymph nodes. The patient’s bone density scan, autoimmune antibodies, bone marrow biopsy, and sacral bone biopsy all came back normal. Imaging test results found no evidence of solid tumors or active TB infection. The patient later recalled having violent coughing prior to the onset of chest pain and several family members having similar symptoms. Her blood sample was sent to the CDC, revealing *Bordetella pertussis* toxin (PT) IgG titer of 110.68 IU/mL. The patient was diagnosed with pertussis and multiple stress fractures from violent coughing. Symptomatic treatments were administered, and the patient’s symptoms improved. The patient was followed up 8 weeks later, she reported no more coughing or chest pain.

**Conclusions:**

Pertussis is not just a pediatric disease, but diagnosis in adults is challenging as patients may present with a myriad of confusing symptoms, such as multiple stress fractures due to violent coughing. Medical and epidemiological histories are key to reaching the correct diagnosis, which is essential for appropriate treatments to avoid further complications. Adult immunization should be suggested both for the protection of the adult population and to prevent transmission to children.

## Background

Pertussis is an infectious respiratory disease caused by the bacterium *Bordetella pertussis*, characterized by paroxysms of severe coughing and the characteristic “whoop”, and predominantly affects children. The global annual incidence of the disease for children under the age of five has been estimated to be 24.1 million, with 160,700 cases of mortalities [1]. The disease is highly contagious, with between 50–90% of individuals contracting the disease after exposure, and affects infants most severely [2]. The incubation period typically lasts 7–10 days and the disease is most contagious 3 weeks after the onset of coughing symptoms.

Adult pertussis patients may present with a wide range of symptoms and complications, such as sinusitis, otitis media, urinary incontinence, pneumonia, weight loss, rib fractures, and syncope [3]. Several cases of adolescent or adult pertussis patients presenting with atypical symptoms have previously been reported, including fracture of the first rib, abdominal wall hematoma, and liver lobe extrusion [4, 5].

Rib fractures in adult pertussis patients are not uncommon, yet fractures in other locations are extremely rare. We report a case of multiple fractures in the ribs, lumbar spine, and sacrum associated with severe coughing caused by *Bordetella pertussis* infection in an adult. This unique case of adult pertussis demonstrates that, though rare, severe bone fracture is a possible presentation of the disease, and the diagnosis should be carefully considered by clinicians.

## Case presentation

A 49-year-old female with a history of gastroesophageal reflux disease (GERD) and chronic atrophic gastritis presented with the main complaint of acute-onset chest pain for 3 weeks. She denied any trauma to the chest, falls, or other injuries prior to the onset of symptoms. She was initially examined elsewhere, where positron emission tomography-computed tomography (PET-CT) revealed multiple fractures in the ribs (right 5^th^-9^th^, left 6^th^-9^th^), the right transverse process of L3, and left side of the sacrum, as well as bilateral pleural effusion, pericardial effusion, right pneumothorax, and enlargement of the left parapharyngeal and subclavian lymph nodes.

**Fig. 1:**
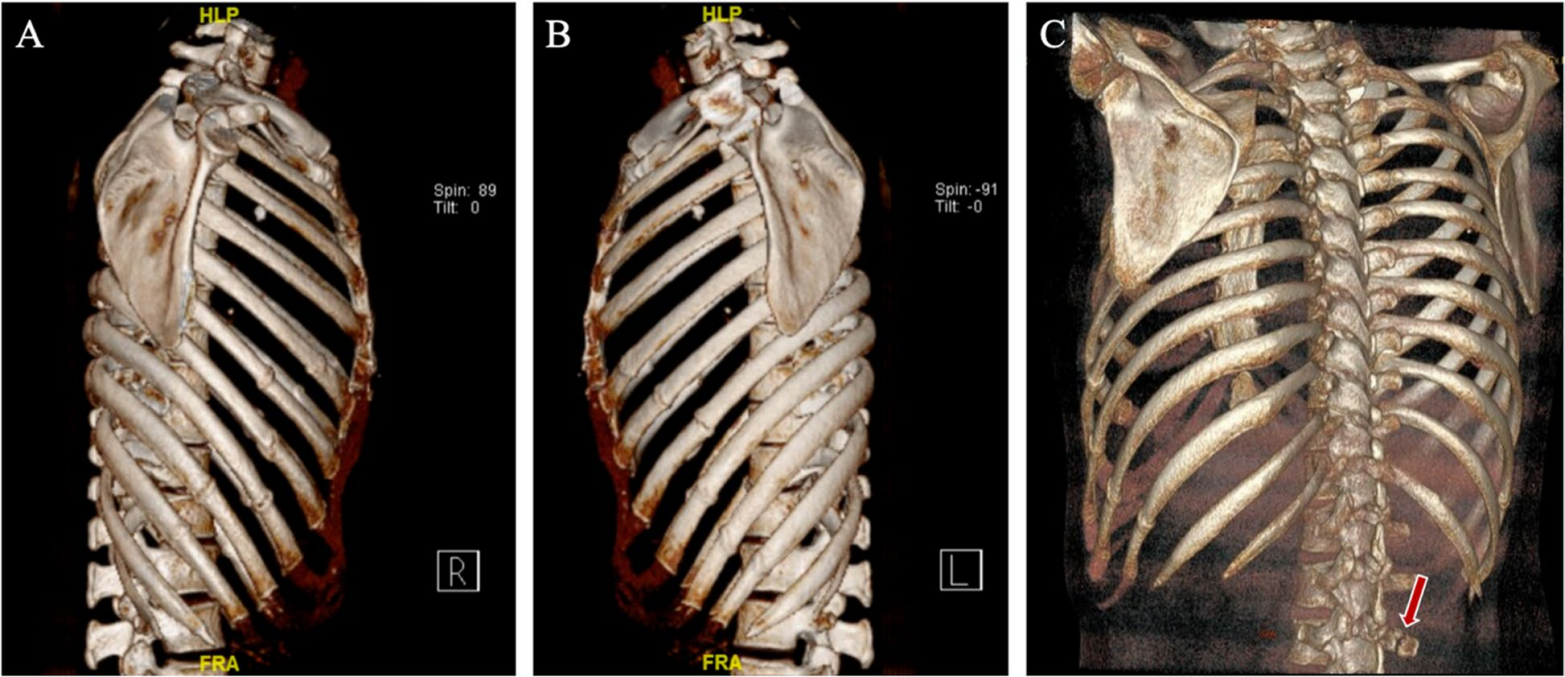
CT reconstruction of the rib cage and vertebrae reveals fractures and formation of calluses. **A** Right rib cage (fractures in 5th-10th). **B** Left rib cage (fractures in 5th-10th). **C** Fracture in the right transverse process of L3. The red arrow indicates the site of fracture

**Fig. 2:**
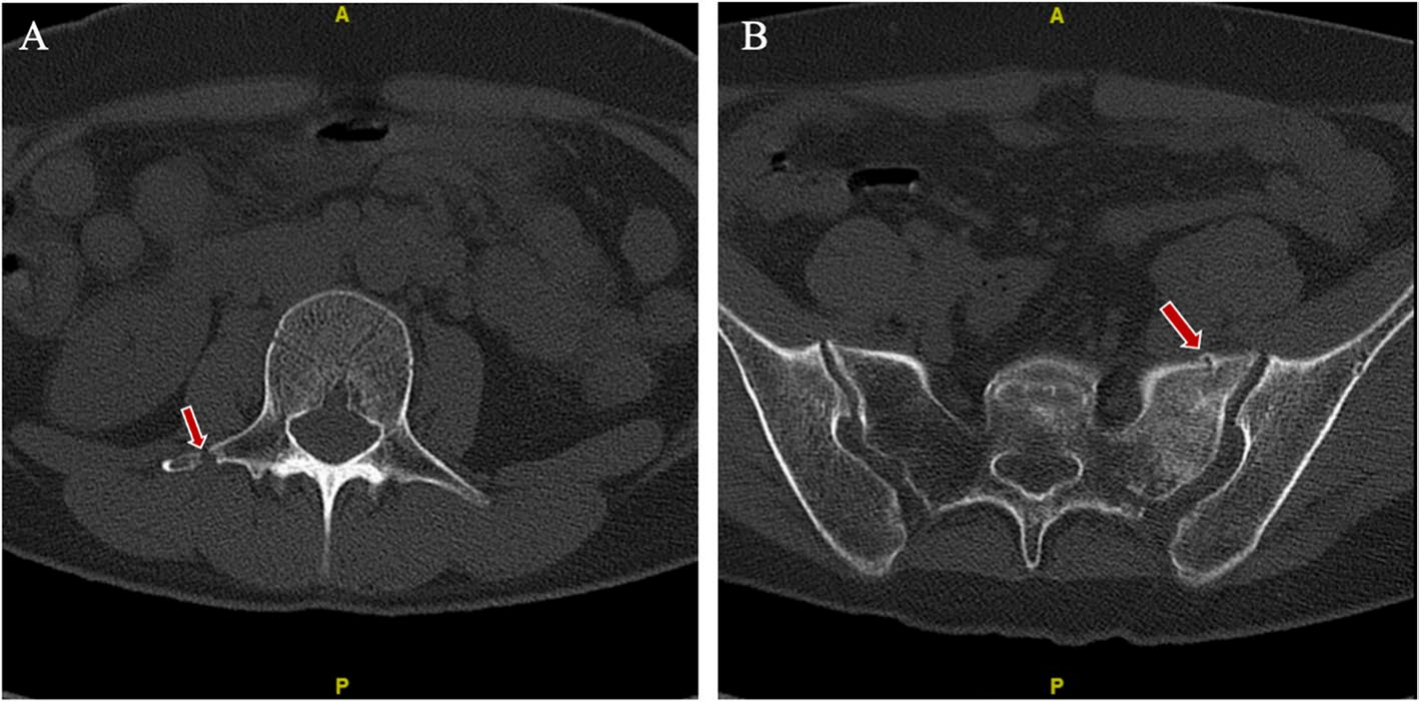
CT scan reveals fractures in L3 and the sacrum. **A** Fracture in the right transverse process of L3. The red arrow indicates the site of fracture. **B** Fracture in the left side of the sacrum. The red arrow indicates the site of cortical discontinuity and abnormal increase in bone density

Upon admission, a general physical examination revealed no significant findings except for tenderness at L3. Blood investigations were unremarkable except for elevated alkaline phosphatase (ALP) of 253U/L, mildly elevated highly sensitive C-reactive protein (hsCRP) of 4.69 mg/L, and a positive T-SPOT.TB test. Chest computed tomography (CT) showed that the bilateral pleural effusion and right pneumothorax have resolved. CT 3D reconstruction of the rib cage and vertebrae showed fractures and formation of calluses in bilateral ribs (right 5^th^-10^th^, left 5^th^-10^th^) and fracture of the right transverse process of L3 (Fig. 1). CT of the lumbar spine and pelvis confirmed fractures in the right transverse process of L3 and the left side of the sacrum (Fig. 2).

Differential diagnoses including osteoporosis, autoimmune disorders, infection, hematological tumors, and primary or metastatic bone tumors were considered. The patient’s bone density scan, autoimmune antibodies, bone marrow biopsy, and sacral bone biopsy all came back normal. Imaging tests found no evidence of solid tumors. Furthermore, imaging results did not indicate active tuberculosis (TB) infection.

The patient then recalled having a violent productive cough accompanied by dyspnea 1 week prior to the onset of chest pain, hence the possibility of stress fractures due to violent coughing was considered. The patient had a previous diagnosis of GERD, which is a common cause of chronic cough due to acid reflux. Hence, a proton pump inhibitor (PPI) was administered, but her symptoms failed to improve.

When further inquired, the patient revealed that her daughter had violent coughing accompanied by fever prior to her illness, while several other relatives recently developed similar symptoms. Hence, the patient’s blood sample was sent to Beijing Center for Disease Control and Prevention (CDC) for testing, revealing *Bordetella pertussis* toxin (PT) IgG titer of 110.68 IU/mL. Blood samples from the patient’s family members were also collected and sent to the CDC (April 2022) (Table 1). A PT-IgG cut-off value of 30 IU/mL has been suggested to indicate recent Pertussis contact, while a titer greater than 100 IU/mL is indicative of active or recent infection with high sensitivity and specificity [6, 7].

**Table 1:** *Bordetella pertussis* toxin (PT) IgG titer of the patient’s relatives who also experienced persistent cough

Relationship to patient	Persistent cough (> 2 weeks)	Cough onset	PT-IgG(IU/mL)
2^nd^ daughter	Yes	2021–12-03	37.78
Self	Yes	2022–01-10	110.68
Mother-in-law	Yes	2022–01-27	156.36
Sister	Yes	2022–02-23	220
1^st^ daughter	Yes	2022–02-24	58.47
Mother	Yes	2022–03-02	9.71

The patient never received the pertussis vaccine as an adult, and she could not recall whether she received the vaccine in childhood. Based on the serological evidence, the patient was diagnosed with pertussis. Since the patient had already entered the convalescent stage of the disease, symptomatic treatments were administered, her symptoms were alleviated, and the patient was discharged. Prescribed medications included montelukast sodium (10 mg q.d.), codeine phosphate (30 mg q.d.), and oxycodone and acetaminophen (5 mg: 325 mg b.i.d.), which were continued for 10 days after discharge. The patient was followed up 8 weeks later, she reported complete resolution of coughing and chest pain and was satisfied with the treatments she received.

## Discussion and conclusions

Rib fractures in adult Pertussis patients are relatively common, affecting up to 4% of patients [2]. Stress fractures in the ribs are common findings in patients suffering from chronic cough and usually occur in the 5^th^-10^th^ ribs [8]. Yet, concurrent stress fractures in the ribs, lumbar spine, and sacrum associated with Pertussis have not been previously reported. Sacral stress fractures can be caused by repetitive axial impact and are mostly unilateral [9]. Violent coughing is often accompanied by involuntary flexion of the vertebral column, which may cause the iliopsoas muscle to exert a repeated external force on the sacrum, ultimately resulting in stress fractures.

The misdiagnosis rate of pertussis in adults is as high as 94.69% and the actual incidence of pertussis in adolescents and adults is significantly underestimated [10]. In fact, more than 5% of adults suffering from chronic cough had serological evidence of pertussis infection, and pertussis may be more common in those with occupational exposure [11]. Our case report highlights the importance of medical and epidemiological histories in the diagnosis of pertussis. Reaching the correct diagnosis is key to prompt treatment and avoiding unnecessary tests and procedures. The patient in our case was treated at multiple medical centers and endured several invasive procedures before the diagnosis was made. Had clinicians been more meticulous in collecting the patient’s epidemiological history, the diagnosis could have been reached much sooner.

In this case, the patient’s 7-year-old daughter, who have been vaccinated in infancy, was the first in the family to contract the disease, transmitting the infection to the patient, who most likely acted as the infection source for other family members. Currently, lifelong vaccine-induced immunity against pertussis is not possible, and the protective effect of the pertussis vaccine decreases over time and typically lasts for about 6 years [12, 13]. Hence, without booster vaccinations, most adolescents and adults are susceptible to pertussis infection.

Furthermore, it has been suggested that adult patients play an important role in the transmission of pertussis, particularly in household outbreaks [14]. Specifically, previous studies have suggested that adults accounted for most infection sources in infant pertussis cases, and vaccinating the parents against pertussis could have prevented a significant proportion of infant infections, which are potentially fatal [15, 16].

*Bordetella pertussis* infection should always be carefully considered and ruled out in adult patients presenting with severe coughing and rib fractures, especially for those with epidemiological histories. More severe bone fractures such as in the lumbar and sacral spine can occur due to violent coughing in pertussis, and therefore is not a reason to overlook the disease. Furthermore, since Tdap vaccination is a safe and effective measure to prevent pertussis, clinicians should routinely recommend vaccination to immunocompromised patients and adults with occupational or household exposure, such as those who work or live with young children [17, 18].

In conclusion, pertussis is not just a pediatric disease, but diagnosis in adults is challenging as patients may present with a myriad of confusing symptoms, such as multiple bone fractures. Medical and epidemiological histories are key to reaching the correct diagnosis, which is essential for appropriate treatments to avoid further complications. Adult immunization should be suggested both for the protection of the adult population and to prevent transmission to children.

## Data Availability

Not applicable.

## Abbreviations

GERD: Gastroesophageal reflux disease
PET-CT: Positron emission tomography-computed tomography
ALP: Alkaline phosphatase
hsCRP: Highly sensitive C-reactive protein
CT: Computed tomography
TB: Tuberculosis
PPI: Proton pump inhibitor
CDC: Center for Disease Control and Prevention
PT-IgG: Pertussis toxin IgG

## References

[1] Yeung KHT, Duclos P, Nelson EAS, Hutubessy RCW (2017). An update of the global burden of pertussis in children younger than 5 years: a modelling study. Lancet Infect Dis.

[2] Decker MD, Edwards KM (2021). Pertussis (Whooping Cough). J Infect Dis.

[3] De Serres G, Shadmani R, Duval B, Boulianne N, Déry P, DouvilleFradet M, Rochette L, Halperin SA (2000). Morbidity of pertussis in adolescents and adults. J Infect Dis.

[4] Prasad S, Baur LA (2001). Fracture of the first rib as a consequence of pertussis infection. J Paediatr Child Health.

[5] Madi MY, Shahwan MY, Nayar C, Kher S (2019). Coughing Up a Lung: A Curious Case of Pertussis. Cureus.

[6] de Melker HE, Versteegh FG, Conyn-Van Spaendonck MA, Elvers LH, Berbers GA, van Der Zee A, Schellekens JF (2000). Specificity and sensitivity of high levels of immunoglobulin G antibodies against pertussis toxin in a single serum sample for diagnosis of infection with Bordetella pertussis. J Clin Microbiol.

[7] Zhang Q, Zheng H, Liu M, Han K, Shu J, Wu C, Xu N, He Q, Luo H (2012). The seroepidemiology of immunoglobulin G antibodies against pertussis toxin in China: a cross sectional study. BMC Infect Dis.

[8] Hanak V, Hartman TE, Ryu JH (2005). Cough-induced rib fractures. Mayo Clin Proc.

[9] Riedl M, Roediger J, Pohlmann J, Hesse J, Warschun F, Wolfarth B, Ueberschär O (2022). Laterality of sacral stress fractures in trained endurance athletes: Are there biomechanical or orthopaedic risk factors?. Sports Orthopaedics and Traumatology.

[10] Huang H, Zhu T, Gao C, Gao Z, Liu Y, Ding Y, Sun J, Guo L, Liu P, Chen D, Wang L, Wu S, Zhang Y (2015). Epidemiological features of pertussis resurgence based on community populations with high vaccination coverage in China. Epidemiol Infect.

[11] Koh MT, Liu CS, Chiu CH, Boonsawat W, Watanaveeradej V, Abdullah N, Zhang X, Devadiga R, Chen J (2016). Under-recognized pertussis in adults from Asian countries: a cross-sectional seroprevalence study in Malaysia. Taiwan and Thailand Epidemiol Infect.

[12] Cherry JD (2014). Adult pertussis in the pre- and post-vaccine eras: lifelong vaccine-induced immunity?. Expert Rev Vaccines.

[13] Schwartz KL, Kwong JC, Deeks SL, Campitelli MA, Jamieson FB, Marchand-Austin A, Stukel TA, Rosella L, Daneman N, Bolotin S, Drews SJ, Rilkoff H, Crowcroft NS (2016). Effectiveness of pertussis vaccination and duration of immunity. CMAJ.

[14] Baptista PN, Magalhães VS, Rodrigues LC (2010). The role of adults in household outbreaks of pertussis. Int J Infect Dis.

[15] Wendelboe AM, Njamkepo E, Bourillon A, Floret DD, Gaudelus J, Gerber M, Grimprel E, Greenberg D, Halperin S, Liese J, Muñoz-Rivas F, Teyssou R, Guiso N, Van Rie A (2007). Infant Pertussis Study Group. Transmission of Bordetella pertussis to young infants. Pediatr Infect Dis J.

[16] de Greeff SC, Mooi FR, Westerhof A, Verbakel JM, Peeters MF, Heuvelman CJ, Notermans DW, Elvers LH, Schellekens JF, de Melker HE (2010). Pertussis disease burden in the household: how to protect young infants. Clin Infect Dis.

[17] Kretsinger K, Broder KR, Cortese MM, Joyce MP, Ortega-Sanchez I, Lee GM, Tiwari T, Cohn AC, Slade BA, Iskander JK, Mijalski CM, Brown KH, Murphy TV, Centers for Disease Control and Prevention, Advisory Committee on Immunization Practices, Healthcare Infection Control Practices Advisory Committee (2006). Preventing tetanus, diphtheria, and pertussis among adults: use of tetanus toxoid, reduced diphtheria toxoid and acellular pertussis vaccine recommendations of the Advisory Committee on Immunization Practices (ACIP) and recommendation of ACIP, supported by the Healthcare Infection Control Practices Advisory Committee (HICPAC), for use of Tdap among health-care personnel. MMWR Recomm Rep.

[18] Xu J, Liu S, Liu Q, Rong R, Tang W, Wang Q, Kuang S, Zhou C (2019). The effectiveness and safety of pertussis booster vaccination for adolescents and adults: A systematic review and meta-analysis. Medicine (Baltimore).

